# Effect of the Game Design, the Goal Type and the Number of Players on Intensity of Play in Small-Sided Soccer Games in Youth Elite Players

**DOI:** 10.1515/hukin-2015-0125

**Published:** 2015-12-30

**Authors:** Joaquín González-Rodenas, Ferran Calabuig, Rafael Aranda

**Affiliations:** 1 Department of Physical Education and Sport. University of Valencia (Spain)

**Keywords:** physiological responses, soccer training, aerobic performance, fitness, football

## Abstract

The aim of this study was to compare the effects of game design modification, the type of the goal and the number of players on the intensity of play in small-sided soccer games (SSGs) in youth elite players. Twenty young soccer players (age 13.7 ± 0.5 years, body mass 57.4 ± 7.8 kg, body height 1.67 ± 7.8 m, maximal heart rate 201.1 ± 8.2 beats/min) performed three types of SSGs (possession play (PP) vs. regular goals (RG) vs. small goals (SG)) in both four-a-side and six-a-side formats. The heart rate responses were recorded and analysed as an indicator of the intensity of play. The four-a-side format obtained higher intensity of play than six-a-side for PP (p<0.05), but not for SG and RG. SG showed higher intensity of play than RG for four-a-side (p<0.001), but not for six-a-side. PP registered higher intensity of play than RG (p<0.05), but not than SG in four-a-side, whereas in six-a-side no differences were found between the three formats. In conclusion, the modification of variables such as the number of players, the game design and the type of the goal influences the intensity of play in small-sided soccer games in youth players.

## Introduction

Improving aerobic fitness can enhance physical performance of soccer players (Helgerud et al., 2001; Hoff et al., 2002). In this context, the preparation of soccer players for competition requires training to be viewed from an ergonomics perspective whereby the training requirements are matched to the competitive demands of match play (Reilly, 2005). For this reason, small-sided games (SSGs) are employed by many amateur and professional teams as an effective tool for aerobic training since they combine technical, tactical and physiological training stimuli (Rampinini et al., 2007). SSGs can illicit heart rate responses around 90 to 95% of the maximal heart rate (HR_max_) (Hoff et al., 2002) and lead to improvements in both aerobic fitness and physical match performance (Helgerud et al., 2001).

SSGs have characteristics defined by factors that offer multiple possibilities of variation. Several studies have analysed the effect of modifying the number of players (Dellal et al., 2011a; Hill-Haas et al., 2009b; Jones and Drust, 2007; Little and Williams, 2007), pitch size (Kelly and Drust, 2009; Tessitore et al., 2006), exercise duration (Casamichana et al., 2012; Fanchini et al., 2011; Hill Hass et al., 2009c), coach encouragement (Rampinini et al., 2007), rule changes (Hill-Haas et al., 2010), ball contacts (Dellal et al., 2011b) and different periods of play (Dellal et al., 2012) on the physiological demands of soccer.

On the other hand, different SSGs’ designs can be used, such as games without space orientation where the main objective is to keep ball possession (possession play), or games with specific space orientation in terms of invasion where the objective is to score in small goal nets without goalkeepers or regular goals with goalkeepers (invasion play). In this context, few studies have analysed the effect of a game design and the type of the goal on the physiological demands of soccer. Sassi et al. (2005) and Mallo and Navarro (2008) using four-a-side and three-aside format, respectively, observed that possession play called for greater physiological demands than the same game with regular goals and goalkeepers. Casamichana et al. (2011) showed that SSGs based on possession play and small goals were more intense than SSGs based on regular goals and goalkeepers. However, Dellal et al. (2008) found for eight-a-side format that possession play was less intense than in a regular game. Therefore, more research is necessary to determine the effects of a game design and the type of the goal on physiological demands during SSGs in soccer.

The aim of this study was to compare the effect of game design modification, the type of the goal and the number of players on the intensity of play in small-sided soccer games (SSGs) in youth elite players.

## Material and Methods

### Participants

Twenty young male soccer players belonging to an academy team from a first-division professional club in Spain participated in the study (age 13.7 ± 0.5 years, body mass 57.4 ± 7.8 kg, body height 1.67 ± 7.8 m, maximal heart rate 201.1 ± 8.2 beats/min). The subjects had broad experience of playing soccer (7.3 ± 0.5 years) and competed at the highest level for their age (under-14s). Three players were excluded from the study as they did not give sufficient data for comparison. The University of Valencia ethics committee approved the research protocol before the beginning of the study and written informed consent was received from all parents after a detailed explanation of the study.

### Measures

To obtain maximal heart rate values for reference purposes, the participants completed a maximum exercise test (Test Leger; Leger et al., 1988). During each SSG their heart rate was recorded every 5 s with a heart rate monitor (Polar Team system, Polar Electro, Finland) and analysed with Polar Pro trainer 5 (Polar Electro, Finland). Individual mean heart rates during the different SSGs were determined in order to obtain an indication of the overall intensity of play. To determine the mean heart rate the first 90 s of each 4 min period were excluded in order to measure the heart rate when it was stabilised. Heart rate data were expressed as a percentage of the maximal heart rate.

### Procedures

SSGs were performed at one or two training sessions per week from April to June 2010. The training sessions took place during the same hours (between 19:00 and 21:00), on an artificial turf surface. Prior to each SSG, a 15 min standardised warm-up took place.

Table 1 shows the main features of the SSGs conducted in this study. Each SSG was supervised by a coach and every time a ball went out of the field it was quickly replaced in order to allow continuity of play. Moreover, coaches encouraged players to put pressure on other players after losing possession or exert pressure after recovering possession and increase the pace of play during each SSG. Each SSG was performed as interval training consisting of six bouts of 4 min duration with 2 min of passive recovery between the bouts. The order in which the SSG formats were performed during the course of the training sessions was randomised. All SSGs had the same area per player (100 m^2^) and followed the same tactical concept.

Three different SSGs were performed: 1) possession play (the aim of the teams was to maintain ball possession), 2) small goals (the aim of the teams was to score a goal, and try not to concede one, using small goals (height: 1 m; width: 2 m)) and 3) regular goals (the aim of the teams was to score a goal, and try not to concede one, using regular goals and goalkeepers (height: 2.44 m; width: 7.32 m)). The three SSG formats were performed by both four-a-side and six-a-side teams.

### Analysis

All results are reported as mean and standard deviations (mean ± SD). Before parametric statistical testing, the normality of the data was verified using the Kolmogorov-Smirnov test. A one-way analysis of variance (ANOVA) followed by a Bonferroni post hoc test was employed to evaluate the differences in intensity of play (dependent variable) according to the game design and the type of the goal (PP vs. SG vs. RG) and the number of players (six-a-side vs. four-aside) (independent variables). The level of statistical significance was set at *p*<0.05. All statistical analyses were carried out with SPSS statistical analysis software (SPSS 15.0, Chicago, Illinois, USA).

## Results

### Effect of the number of players

**Figure 1:**
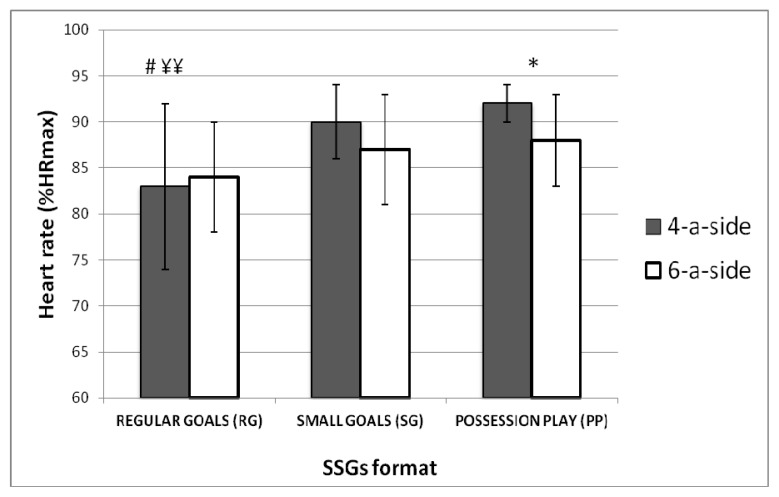
Average ± SD heart rate responses according to SSG format and the number of players. *p<0.05 comparing four-a-side with six-a-side within the same SSG format. #p<0.05 compared with small goals. ¥¥ p<0.001 compared with possession play.

Figure 1 shows that PP in four-a-side format registered higher intensity of play and a smaller standard deviation (92±2% HR_max_) than PP in six-a-side format (88±5% HR_max_) (*p*<0.05).

No significant differences were found for the SG format between four-a-side (90±4% HR_max_) and six-a-side (87±6% HR_max_). Also, no significant differences were found for the RG format between four-a-side (83±9% HR_max_) and six-a-side (84±6% HR_max_).

### Effect of the game design and the type of the goal

Figure 1 shows that SG accounted for significantly higher intensity of play than RG for four-a-side (90±4% vs. 84±6% HR_max_) (*p*<0.05), but not for six-a-side (87±6% vs. 84±6% HR_max_), respectively.

Moreover, Figure 1 shows that PP in four-aside format obtained significantly higher intensity of play than RG for four-a-side (92±2%HR_max_ vs. 83±9%HR_max_) (*p*<0.01), but not for six-a-side (88±5%HR_max_ vs. 84±6%HR_max_), respectively.

No significant differences were found between PP and SG in four-a-side or six-a-side formats.

Remarkable differences were observed in standard deviations between formats. RG in four-a-side games registered the highest SD compared with the same format in six-a-side games and compared with smalls goals and possession play.

## Discussion

The main outcome of this study was that modifying the number of players, a game design and the type of goals affects the intensity of play in small-sided soccer games.

Regarding the effects of the variable number of players, this study found that four-aside games obtained significantly higher intensity of play than six-a-side in PP, but not in SG or RG. These results are different from those found by Rampinini et al. (2007) and Hill-Hass et al. (2009b) who observed that four-a-side was more intense than six-a-side in SSGs with small goals. However, with respect to SSGs with regular goals, Jones and Drust (2007) and Dellal et al. (2008) did not observe differences in intensity of play between four-a-side and eight-a-side games. Nevertheless, Katis and Kellis (2009) found that SSGs with three-a-side induced higher intensity of play than six-a-side with regular goals. In this context, not finding differences between four-aside and six-a-side in SSGs with regular goals in this study may be because the four-a-side format means the total playing space is reduced and therefore, the goals are closer to each other, so that more shooting opportunities are created. Consequently, players may have less contact with the ball in terms of passes, dribbles or tackles designed to reach the goal in comparison with six-a-side where reaching the goal needs more game elaboration. As higher standard deviation indicated (83±9%HR_max_), the intensity was more variable among players in RG for the four-a-side game and no differences were found in the six-aside game.

With regard to the effect of the type of the goal on intensity of play, this study observed that for four-a-side games, SG registered higher intensity of play than RG. However, no differences were found for six-a-side games. In this context, Casamichana et al. (2011) found that SSGs in four-a-side played with small goals were more intense than in the same game played with regular goals. However, Castellano et al. (2013) did not find differences between small and regular goals in seven-a-side game format. These results may indicate that the effect of the type of the goal on the intensity of play is higher for SSGs with few players (four-a-side) than those performed with many players (six-a-side or more). It could result from the fact that for the four-a-side format the use of regular goals and their proximity allow players to shoot at the goal easily without the necessity of performing numerous technical elements with the ball to achieve a good shooting position. However, the use of small goals changes this situation, as a larger number of activities with the ball per player and greater game elaboration per team are required to achieve an appropriate shooting opportunity and therefore, small goals register higher intensity of play than regular goals. On the other hand, the effect of the type of the goal in the six-a-side game may be non-significant compared with the four-a-side game considering that the larger field and the greater distance between goals may require greater game elaboration per team to achieve an appropriate shooting opportunity regardless of the type of the goal and as a consequence no differences were found between small goals and regular goals in terms of intensity of play for six-a-side games.

With respect to the effect of the game design, this study found that PP in four-a-side registered higher intensity of play than RG, but not SG, whereas in six-a-side no differences were found between the three different game types. Casamichana et al. (2011) showed that SSGs based on possession play and small goals were more intense than SSGs based on regular goals and goalkeepers in four-a-side games. Sassi et al. (2005) observed that SSGs with goalkeepers decreased physiological responses compared with possession play in four-a-side. Furthermore, Mallo and Navarro (2008) observed for three-aside games that the distance covered, intensity of play, ball contacts and short passes decreased in games with regular goals and goalkeepers in comparison with possession play. However, Dellal et al. (2008) observed in eight-a-side games with regular goals and goalkeepers higher intensity of play than possession play, although the space per player (168m^2^/player) was different from this study. The authors considered that players were more motivated in the regular goal games and therefore, registered higher intensity than in possession play. Also, a recent study conducted by Castellano et al. (2013) did not find any differences between possession play, regular goals and goalkeepers and small goals in seven-aside games.

These findings suggest that a game design may be important in terms of affecting the intensity of play in SSGs when possession play is compared with games using regular goals and goalkeepers in games with few players (three-aside and four-a-side games), but this variable does not have as much effect on SSGs when possession play is compared with small goals in any format or regular goals and goalkeepers in formats with many players (six-a-side, seven-a-side and eight-a-side). This could be because in SSGs with many players the ball contacts per player decrease with respect to SSGs with few players (Jones and Drust, 2007; Katis and Kellis, 2009) and the effects of game variables may be less effective in producing changes in intensity of play.

Another finding of this study was that the average heart rate ranged from 83±9%HR_max_ in four-a-side with RG to 92±2HR_max_ in four-a-side with PP. These ranges of intensity are similar to those in previous studies (Little and Williams, 2006; Rampinini et al., 2007; Sassi et al., 2005) and higher than in some other studies (Casamichana et al., 2011; Hill-Haas et al., 2009b; Katis and Kellis, 2009) in which the average heart rate ranged between 86 and 92% of the HR_max_ and 82 and 89% of the HR_max_, respectively. Therefore, SSGs carried out in this study may be used to improve maximum oxygen uptake (VO_2máx_) (Helgerud et al., 2001; Hoff et al., 2002; Impellizzeri et al., 2006).

The main limitation of this study is that evaluating the heart rate as the sole indicator of the intensity of play means that only the physiological characteristics of SSGs were taken into account. The evaluation of lactate concentration or percentage of play in particular zones of intensity calculated individually in relation to VO_2max_ would increase the reliability of data. Also, evaluating the distance covered as well as the number of technical elements would increase the precision of the data presented.

In conclusion, our findings show that the use of regular goals decreases the intensity of play in comparison with the use of small goals or possession play for four-a-side games. Moreover, the four-a-side game shows higher intensity of play than the six-a-side game for possession play, but not for invasion games. Thus, this study shows that soccer coaches should take into account variables such as a game design, the goal type and the number of players in order to design small-sided soccer games and modulate the intensity of play according to specific training objectives.

## Figures and Tables

**Table 1:** Small-sided game formats

Player number	Game design	Type of goal	Area per player	Time (work: passive recovery)	Tactical concept	Encouragement
Four-a-side	Invasion play	Small goals	100m^2^	4:2	Pressure after losing possession	Yes
		Regular goals				
	Possession play	-------------				
Six-a-side	Invasion play	Small goals			Exert pressure after recovering possession	
		Regular goals				
	Possession play	-------------				
